# Impact of Food Components on in vitro Calcitonin Gene-Related Peptide Secretion—A Potential Mechanism for Dietary Influence on Migraine

**DOI:** 10.3390/nu8070406

**Published:** 2016-07-01

**Authors:** Margaret Slavin, Julia Bourguignon, Kyle Jackson, Michael-Angelo Orciga

**Affiliations:** 1 Department of Nutrition and Food Studies, George Mason University, 4400 University Drive, MS 1F8, Fairfax, VA 22030, USA; jbourgu@gmail.com (J.B.); kjacks21@masonlive.gmu.edu (K.J.); 2 School of Systems Biology, George Mason University, 4400 University Drive, MS 3E1, Fairfax, VA 22030, USA; michaelangelo.orciga@gmail.com

**Keywords:** calcitonin gene-related peptide, migraine, food trigger, calcium, grape pomace, ginger, butterbur, petasin

## Abstract

Calcitonin gene-related peptide (CGRP) is a pivotal messenger in the inflammatory process in migraine. Limited evidence indicates that diet impacts circulating levels of CGRP, suggesting that certain elements in the diet may influence migraine outcomes. Interruption of calcium signaling, a mechanism which can trigger CGRP release, has been suggested as one potential route by which exogenous food substances may impact CGRP secretion. The objective of this study was to investigate the effects of foods and a dietary supplement on two migraine-related mechanisms in vitro: CGRP secretion from neuroendocrine CA77 cells, and calcium uptake by differentiated PC12 cells. Ginger and grape pomace extracts were selected for their anecdotal connections to reducing or promoting migraine. *S*-petasin was selected as a suspected active constituent of butterbur extract, the migraine prophylactic dietary supplement. Results showed a statistically significant decrease in stimulated CGRP secretion from CA77 cells following treatment with ginger (0.2 mg dry ginger equivalent/mL) and two doses of grape pomace (0.25 and 1.0 mg dry pomace equivalent/mL) extracts. Relative to vehicle control, CGRP secretion decreased by 22%, 43%, and 87%, respectively. *S*-petasin at 1.0 μM also decreased CGRP secretion by 24%. Meanwhile, *S*-petasin and ginger extract showed inhibition of calcium influx, whereas grape pomace had no effect on calcium. These results suggest that grape pomace and ginger extracts, and *S*-petasin may have anti-inflammatory propensity by preventing CGRP release in migraine, although potentially by different mechanisms, which future studies may elucidate further.

## 1. Introduction

Migraine is a complex primary headache condition that affects 17% of American women and 5.6% of men annually [1]. Worldwide, migraine is responsible for half of all neurological disease-induced disability [2]. Suggestions abound regarding the potential connection between food and migraine, but valid scientific evidence on the subject remains limited.

The current pathophysiological understanding of migraine entails over-sensitization of the brain, which produces a painful response to otherwise normal stimuli, in combination with an inflammatory response. A key inflammatory mediator in neurogenic inflammation of migraine is calcitonin gene-related peptide (CGRP). The connections of CGRP to migraine are numerous. Fibers originating in the trigeminal ganglion terminate in the dura where they release CGRP, and this connection is thought to be central to the genesis of migraine pain [3]. Elevated CGRP levels have been detected in the serum of migraineurs [4,5] and at higher levels in individuals with chronic migraine than in episodic migraineurs [6]; CGRP is capable of precipitating migraine in prone individuals [7]; and sumatriptan, a pharmaceutical intervention used to abort migraine, has been shown to decrease CGRP levels concomitant with symptom relief [8]. Sumatriptan is primarily classified as a 5-HT_1_ (serotonin) receptor agonist, but has also demonstrated inhibition of action potential signaling by inhibiting N-type Ca^2+^ channels in CGRP fibers [3]. Calcium influx upon depolarization is a fundamental signaling mechanism which, among a host of other functions, stimulates the release of CGRP.

Several lines of evidence indicate that food intake may be capable of altering CGRP levels [9,10,11,12], suggesting a potential mechanistic link between diet and migraine whereby some component of food may modulate expression and/or release of CGRP. Conversely, intraperitoneal administration of CGRP is also shown to induce a short term reduction in food intake likely resulting from cAMP/PKA (cyclic adenosine monophosphate/protein kinase A) pathway activation [13]. Foods or components whose ingestion results in a decrease of CGRP would be hypothesized to reduce migraine, whereas those which increase CGRP would be expected to increase migraine.

This project sought to improve our understanding of the potential mechanisms by which grape pomace and ginger may impact migraine. These foods were chosen because of anecdotal connections to migraine. Ginger is renowned for its anti-inflammatory abilities, while anecdotal evidence and traditional medicine connect it with anti-migraine abilities. Grape is viewed suspiciously because of the common belief that red wine triggers migraine events, while the naturally present phenolic compounds have anti-inflammatory qualities. Meanwhile, we have also included a pure compound in our investigations: *S*-petasin is believed to be an active component in the butterbur dietary supplement, which is classified as having “established efficacy” for the prophylaxis of migraine, as reviewed by the American Academy of Neurology and the American Headache Society [14]. Nonetheless, the potential of these foods and supplement for impacting biomechanisms related to migraine have not been sufficiently investigated. We have prepared extractions of ground dried ginger root and two varieties of grape pomace, and tested purified *S*-petasin and these extractions in cell culture models to investigate their ability to modulate CGRP secretion and calcium signaling.

## 2. Materials and Methods

### 2.1. Materials

Solvents—methanol, ethanol, acetone, DMSO (dimethyl sulfoxide), and ultrapure water—and syringe filters were obtained through Fisher Scientific (Pittsburgh, PA, USA). Cell culture media and reagents were purchased from Life Technologies (Grand Island, NY, USA), including fetal bovine serum, horse serum, trypsin-EDTA (ethylenediaminetetraacetic acid), HEPES (4-(2-hydroxyethyl)-1-piperazineethanesulfonic acid) buffer, HBSS (Hank’s balanced salt solution) buffer, penicillin-streptomycin, and Lipofectamine 2000. Poly-d-lysine for cell culture adherence was produced by MP Biomedicals (Santa Ana, CA, USA). Methylene blue, glutaraldehyde, Type I Collagen, nerve growth factor, and *S*-petasin (>98% pure) were obtained through Sigma Aldrich (St. Louis, MO, USA). CGRP enzyme immunoassay kits were produced by Bertin Pharma (Montigny le Bretonneux, France) and purchased through Cayman Chemicals. The GCaMP5 plasmid was provided courtesy of Dr. Nadine Kabbani lab at George Mason University, Fairfax, VA, USA. GCAMP5 is a genetically encoded calcium indicator, originated by Janelia Research (Ashburn, VA, USA). Grape pomace samples from Chrysalis Vineyards (Middleburg, VA, USA) were obtained through Dr. John Parry, formerly at the Agricultural Research Station at Virginia State University (Petersburg, VA, USA). Dried, sliced ginger root was obtained commercially through Penzeys Spices (Wauwatosa, WI, USA).

### 2.2. Extractions

Dried, sliced ginger root was ground in a standard coffee grinder to pass through a 40-mesh sieve, to select for particles of size 0.420 mm or smaller. Ground ginger was extracted at a ratio of 1 g in 10 mL 100% methanol [15]. After 24 h, the combination was centrifuged, liquid extract transferred to a clean tube, and the extraction was repeated twice. The three fractions were combined, filtered with a 0.45 μm nylon syringe filter and evaporated under nitrogen gas stream. Each extraction was performed in triplicate. Average percent yield by weight was 13.1% ± 0.2%.

Two varieties of dried grape pomace (*Tinta cao* and *Cabernet franc*) were ground in a standard coffee grinder to pass through a 40-mesh sieve, to select for particles of size 0.420 mm or smaller. Ground pomace was extracted at a ratio of 1 g in 10 mL solvent (50:50, acetone:water, *v*/*v*) [16,17]. Tubes were vortexed to mix. After 24 h, the combination was centrifuged and the liquid extract transferred to a clean tube, and partially evaporated under nitrogen gas stream, followed by lyophilization to remove water. Each extraction was performed in triplicate.

Dried extracts were stored at −20 °C until further use. Immediately prior to use, extracts were dissolved in DMSO for cell treatments. For cell assays, concentrations of treatments are expressed as mg equivalents of original pomace extracted per mL of cell treatment media (mg PE/mL) or mg dry ginger extracted per mL of cell treatment media (mg PE (pomace equivalent)/mL or mg equivalent/mL).

### 2.3. Cell Culture Maintenance

The CA77 rat medullary thyroid carcinoma cell line is an in vitro model used to study the modulation of CGRP levels. CA77 cells are neuroendocrine, having originated from the neural crest and retaining neuronal tendencies. Specifically, the cells express a high ratio of CGRP mRNA (90%) relative to the alternately spliced calcitonin mRNA. CA77 cells were received from Dr. Andrew Russo, Department of Molecular Physiology and Biophysics, University of Iowa, Iowa City, IA, USA. CA77 cells are now available through the ATCC repository (Manassas, VA, USA) as product number CRL-3234. Cells were maintained on laminin-coated plates with a DMEM-F12 (Dulbecco’s Modified Eagle Medium-Nutrient Mixture F-12) base media, containing 10% fetal bovine serum, 10 mM HEPES, and 50 units/mL penicillin/streptomycin. CA77 cells were passaged via trypsin-EDTA incubation. 

Meanwhile, PC-12 cells are rat adrenal pheochromocytoma cells used as a model for sympathetic neurons due to the presence of Ca^2+^ transporters. PC-12 cells are available for purchase from the ATCC repository (Manassas, VA, USA) as product number CRL-1721. PC-12 cells were maintained on collagen-coated plates with a low-glucose DMEM base media, containing 10% horse serum, 5% fetal bovine serum, 10 mM HEPES, and 50 units/mL penicillin/streptomycin. PC-12 cells were passaged by the cell scraping technique. All cells were maintained under humidity at 37 °C, 5% CO_2_.

### 2.4. Cytotoxicity Assay

To determine appropriate doses for further testing, several doses of the grape extracts and spice extracts were tested for cytotoxicity via the Methylene Blue Assay according to a previously published protocol [18]. In short, cells were exposed to treatments for 24 h in a 96-well plate, after which the viable cells were fixed to the plate with glutaraldehyde and dyed with methylene blue. Subsequently, the plate was rinsed and developed by elution of the dye in ethanol. Absorbance at 570 nm was interpreted as representing a linear relationship with the number of viable cells.

### 2.5. CGRP Assay

CGRP secretion was measured in CA-77 cells according to a method previously reported, with modifications [10]. Cells were seeded in a 96-well, Advanced Tissue Culture coated plates (Greiner Bio-One, Monroe, NC, USA) at 70,000/well. After 24 h, media was removed by gentle aspiration and replaced with treatment solution for 1 h. All treatment solutions, including vehicle control contained 0.1% DMSO. Treatment was immediately followed by incubation with 50 mM KCl to stimulate release of CGRP. After 1 h, media was collected, diluted in buffer as necessary, and assayed for CGRP content using a commercial enzyme immunoassay (EIA) kit, according to manufacturer instructions (Bertin Pharma, Montigny le Bretonneux, France). Samples were diluted with assay buffer as needed to fall within the standard curve. Absorbance readings were taken at 410 nm on a Spectramax M3 multimode plate reader (Molecular Devices, Sunnyvale, CA, USA).

### 2.6. Calcium Influx

We used the transfected probe, GCaMP5, for measuring cytosolic calcium. The probe was transfected into PC-12 cells using lipofection via incubation of cells in the presence of GCaMP5-encoding plasmid and Lipofectamine 2000 reagent for 6 h, according to manufacturer instructions. Following transfection, cells were seeded on a poly-d-lysine coated glass-bottom, black-sided 96-well plate and incubated in regular growth media with 100 ng/mL nerve growth factor (NGF) for 48 h, to induce neuronal differentiation prior to stimulation and imaging. A Zeiss fluorescence microscope was used for imaging at room temperature with GFP (green fluorescent protein) filter with excitation/emission at 470/525 nm. After 48 h, NGF-containing media was removed by careful aspiration, and wells were immediately treated with 100 μL media with extract for 10 min prior to the calcium challenge. Fluorescence readings were then taken every 70 millieseconds for 50 s. After approximately 10 s of baseline readings, cells were challenged with KCl injection while readings continued, to a final concentration of 50 mM KCl. Image sequence data was processed using PhysImage, a fork of Image J open-source image processing software (imagej.nih.gov), manually selecting the cell body as the area of interest and using the Moving Average Filter set to a 5 frame average. A plot of change in fluorescence compared to baseline (ΔF/F_0_) versus time was produced. The maximum ΔF/F_0_ value, typically reached as a spike within the first 5 s after KCl injection, was used as an indicator of the extent of calcium influx upon KCl stimulation, and thus the treatment impact on calcium permeability. Vehicle controls were performed for each 96-well plate.

### 2.7. Statistical Analysis

Statistical analysis was conducted with SPSS for Windows (version 22, SPSS Inc., Chicago, IL, USA). Data in figures is reported as mean ± standard deviation. Differences in means were detected using one-way ANOVA and either Tukey’s (dose comparison) or Dunnett’s (comparison only to vehicle control) post hoc test. Statistical significance was defined at *P* ≤ 0.05.

## 3. Results

### 3.1. Cytotoxicity Assay

**Figure 1:**
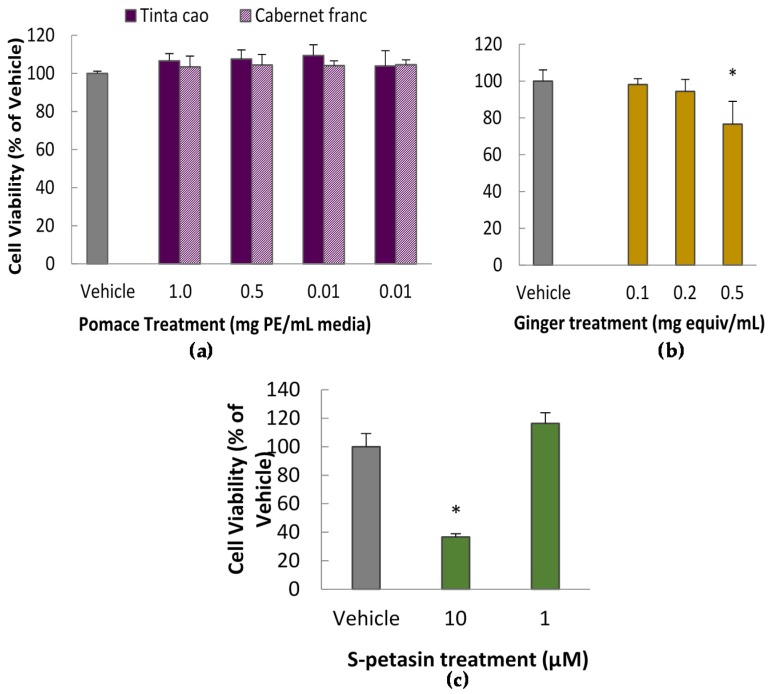
CA77 cell viability after 24-h treatment with extracts: (**a**) *Tinta cao* and *Cabernet franc* grape pomace extract treatments (*n* = 8), (**b**) Ginger root extract treatments (*n* = 8), and (**c**) purified *S*-petasin treatments (*n* = 4). PE = pomace equivalents. Results obtained via the Methylene Blue Cytotoxicity Assay. All treatments contain 0.1% DMSO (dimethyl sulfoxide), including vehicle. * Indicates a value statistically different from the vehicle (*P* < 0.05).

The cytotoxicity assay successfully identified grape pomace extract, ginger extract, and purified *S*-petasin treatment doses which did not impact cell viability, and thus were acceptable for further testing. Results of the grape pomace cytotoxicity assay with CA-77 cells show that all doses of extracts at or below 1.0 mg equivalents/mL media did not impact cell viability (*P* < 0.05), as compared to the vehicle control (Figure 1). Higher doses could not be tested due to extract solubility and a DMSO solvent maximum of 0.1% total volume in the treatment media. In other words, grape pomace extracts were not toxic to cultured CA-77 cells at achievable doses. Results in PC-12 cells with solvent extractions followed the same pattern.

Meanwhile, ginger extract treatment doses at or below 0.2 mg equivalents/mL did not impact cell viability, and *S*-petasin treatments at 1 μM also produced no statistical difference. Higher doses of both treatments were observed to reduce cell viability (Figure 1b,c).

### 3.2. CGRP Secretion

**Figure 2:**
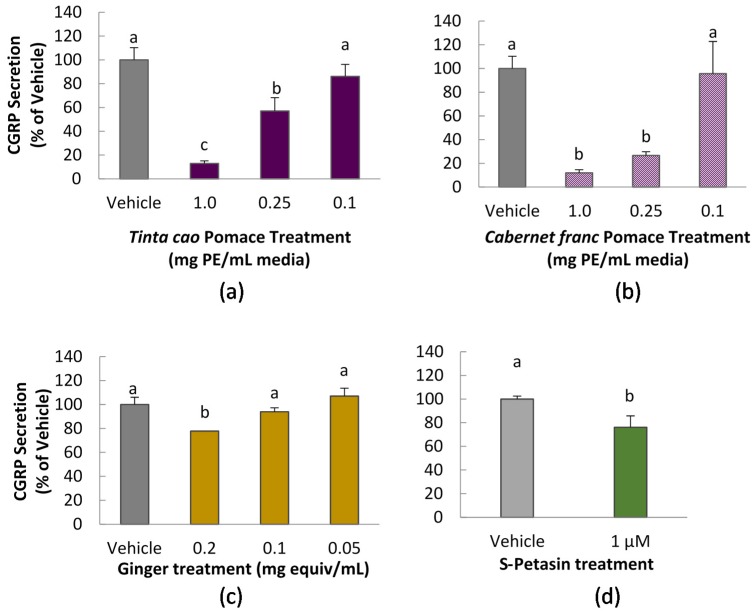
CGRP (Calcitonin gene-related peptide) secretion levels upon treatment with extracts: (**a**) *Tinta cao* grape pomace extract (*n* = 6), (**b**) *Cabernet franc* grape pomace extract (*n* = 6), (**c**) dried ginger root extract (*n* = 9), and (**d**) purified *S*-petasin (*n* = 4). CA-77 cells were treated for 1 h with extracts at specified final doses. All treatments contain 0.1% DMSO, including vehicle. Results obtained via commercial ELISA kit. Columns of the same sub-figure marked by the same letter are not statistically different (*P* < 0.05).

The CGRP secretion assay demonstrated that grape pomace extracts, ginger extract, and purified *S*-petasin could inhibit CGRP secretion in CA77 cells at various doses. The high and medium dose of both grape pomace extracts produced a significant decrease in CGRP secretion by CA77 cells, upon a 1 h pre-treatment with the extracts. At a dose of 1.0 mg pomace equivalent/mL treatment, the *Tinta cao* extraction resulted in an 87% decrease in CGRP secretion (shown in Figure 2a), which was statistically significant as compared to vehicle control and the lower doses of *Tinta cao* extraction (*P* < 0.05). Varying the concentration produced a dose dependent response, where the medium dose of 0.25 mg PE/mL had approximately half the effect of the high dose, reducing CGRP secretion by 43%. The low *Tinta cao* dose of 0.1 mg PE/mL did not produce a significant change, as compared to the vehicle. *Cabernet franc* extraction treatments produced a similar response, decreasing CGRP secretion by 88% and 73% for the high and medium doses, respectively (Figure 2b). The lowest dose of *C. franc* did not produce a statistical difference in CGRP secretion.

Upon 1 h pre-treatment with extracts, the high dose of ginger extract produced a significant decrease in CGRP secretion by CA77 cells. At a dose of 0.2 mg equivalent/mL treatment, the ginger methanol extraction resulted in a 22% decrease in CGRP secretion (shown in Figure 2a), which was statistically significant as compared to vehicle control and the lower doses of ginger methanol extracts. *S*-petasin was tested at only one dose of 1.0 μM, which did produce a significant decrease in CGRP secretion by 24%, as compared to the 0.1% DMSO vehicle.

### 3.3. Effect on Calcium Influx

**Figure 3:**
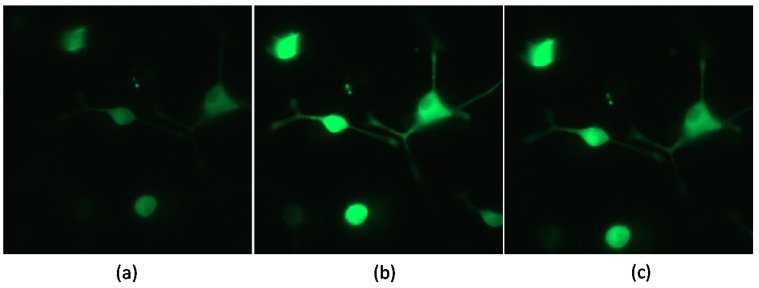
Time lapse of calcium-mediated fluorescence following KCl stimulation. Images depict the fluorescence in GCaMP5 transfected PC-12 cells pre-treated with 0.1% DMSO Vehicle. Images were taken (**a**) before, (**b**) immediately after, and (**c**) 20 s post-KCl injection. Final KCl concentration = 50 mM.

The impact of treatments on acute KCl-stimulated calcium influx was measured using the fluorescent, genetically-encoded GCaMP5 calcium indicator and fluorescence microscopy. The data shows no statistical difference in maximum fluorescence between grape pomace treatment or the vehicle control (Table 1). Visual images of the observed progression before, immediately after, and 20 s after KCl stimulation are shown in Figure 3.

Given that no difference was detected for grape pomace extracts, it is expected that calcium channels remained unobstructed in the presence of these treatments, thus allowing calcium influx into the cell. Therefore, observed decreases in CGRP secretion by grape pomace extracts cannot be attributed to modulation of calcium signals.

**Figure 4:**
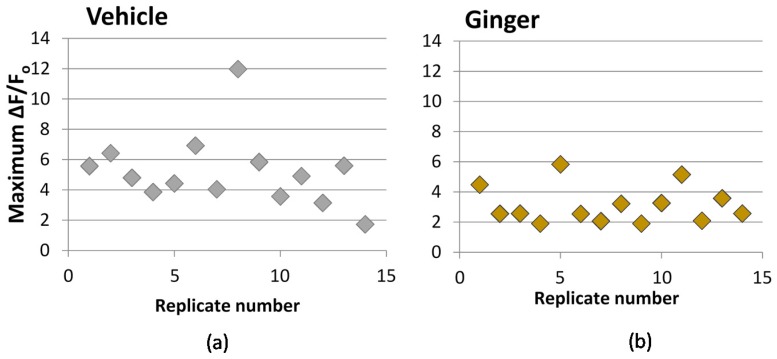
Calcium influx following treatment with methanol ginger extract. PC-12 cells transfected with GCaMP5 fluorescent calcium indicator were pre-treated for 10 min with (**a**) 0.1% DMSO vehicle or (**b**) 0.2 mg equiv/mL ginger extract. Maximum change in fluorescence compared to background (ΔF/F_o_) when stimulated with 50 mM KCl is reported. All treatments contain 0.1% DMSO in media, including vehicle. (*P* = 0.007, *n* = 14).

The impact of ginger extract on calcium influx is shown in Figure 4. When a decrease of fluorescence is observed as compared to vehicle control—as is seen with the ginger extract (*P* = 0.007, *n* = 14)—it is expected the treatment is blocking one or more type of calcium channel.

## 4. Discussion

This study explored the impact of grape pomace and ginger extracts, and purified *S*-petasin on two mechanisms related to migraine inflammation: the release of CGRP by CA-77 neuroendocrine cells and calcium signaling in PC-12 cells. CGRP and its signaling properties are key targets for drug discovery for migraine, thus the ability of food to impact CGRP is of high interest.

Two mechanisms have been observed to cause cellular release of CGRP in trigeminal cells: calcium signal and low pH [19]. In this study, we verified the pH of each prepared cell media plus extract to be approximately 7.2. Interestingly, grape pomace extracts displayed the strongest ability to inhibit CGRP secretion of any of the tested substances. This is notable not only for the scale of the inhibition, but also because the grape pomace extracts did not significantly inhibit calcium uptake upon stimulation. This suggests that inhibition of CGRP release of grape pomace extracts occurs by a mechanism different from calcium channel inhibition. We did not explore the chemical composition of grape pomace extracts in this study, but the phenolic composition of *Tinta Cao* extracts prepared by this extraction procedure is previously reported, and chlorogenic acid was the predominating phenolic acid, followed by ferulic, vanillic, and p-coumaric acids [17]. Total phenolics content was reported at 72.0 mg gallic acid equivalents/g pomace. Presumably, as red grapes, both pomace extracts would also contain anthocyanins, procyanidins, flavonols, and catechins [20]. It is reasonable to propose these phenolic acids and polyphenolics may be responsible for the activity witnessed here.

Cocoa extracts, which are also known to contain high levels of phenolic compounds, showed a similarly dramatic suppression of CGRP secretion, both in cell culture and an in vivo rat feeding study [10,11]. Interestingly, the authors suggest that calcium channel blocking is the likely mechanism, having shown that the cocoa extracts blocked effects of KCl stimulated calcium influx. However, food phenolic bioactivity is an intensely researched area, but no evidence could be found in the literature of calcium channel blockade activity by cocoa phenolics or more generally, cocoa.

Meanwhile, ginger extract at the highest tested dose of 0.2 mg ginger equivalents/mL media demonstrated a mild decrease in calcium uptake as well as a mild reduction in CGRP secretion. While we did not assess composition of the ginger extract, a prior study demonstrated that extracts prepared by this method contain 6-, 8-, and 10-gingerol, as well as 6-shogoal [15]. The proportion of shogoals, the dehydrated form of gingerols, is increased in dried ginger root as compared to fresh, thus we might expect higher shogoals in our dried ginger extracts [21]. The gingerol family of compounds has been identified as calcium channel antagonists [22]. The minor decrease in CGRP secretion may have been mediated by a relative decrease in the intracellular calcium signal caused by the gingerols’ calcium channel antagonism. Ginger has been recognized for centuries in traditional medicine for its anti-inflammatory and analgesic properties, which is increasingly corroborated by demonstrations of impact on cell signaling in various models, including inhibition of prostaglandin and leukotriene synthesis, and inhibition of cyclooxygenase-1 (COX-1) and inducible nitric oxide synthase (iNOS) enzyme activity [21]. Interestingly, a randomized placebo-controlled trial successfully relieved acute migraine symptoms with a sublingual feverfew and ginger preparation [23].

In the present study, CGRP secretion from CA-77 cells was mildly but statistically significantly reduced by 1 μM *S*-petasin treatment. Treatment with *S*-petasin also significantly decreased the calcium influx in PC-12 cells. Thus, it is likely that the inhibition of calcium uptake is at least partially responsible for the decrease in CGRP secretion witnessed here. *S*-petasin, an eremophilane sesquiterpenoid, and other petasins are the suspected bioactive components of butterbur (*Petasites hybridus*) extract, the only dietary supplement with Level A “established efficacy” evidence for the prophylaxis of migraine, as reviewed by the American Academy of Neurology and the American Headache Society [14]. It is a known antagonist of l-type voltage-gated calcium channels and has antispasmotic activity at least partially independent of this calcium blocking ability [24,25,26,27]. Impact of *S*-petasin or butterbur on CGRP levels has not been previously reported in vitro or in vivo.

*Petasites* extract, though not specifically petasins, has been demonstrated to inhibit leukotriene synthesis and COX-2 expression in cell models [28]. Petasins other than *S*-petasin have demonstrated ability to inhibit leukotriene synthesis [29]. Thus, reductions in CGRP are only one potential mechanism to explain the anti-migraine activity of the supplement. Importantly, the *Petasites hybridus* plant contains hepatotoxic, mutagenic, and carcinogenic pyrrolizidine alkaloids. Unpurified botanical supplements (i.e., leaves or crude extracts) should be avoided due to these potentially severe side effects. Purified *Petasites* extracts are available, with higher concentrations of the active petasins, and concentrations of toxic alkaloids below 0.1 ppm [30].

There is some in vivo evidence in the literature to support the notion that diet is capable of impacting CGRP levels, both in circulation and specifically in the brain. One study demonstrated that rats orally fed grape seed extract for 14 days had lower basal expression of CGRP in the neurons and microglia of the trigeminal nucleus caudalis than control rats [12]. Furthermore, there is believed to be shared pathology between TMJ disorders and migraine. When rats were injected with Freud’s adjuvant to stimulate the tempromandibular joint (TMJ) capsule, those who received the oral grape seed extract experienced repressed levels of phosphorylated-p38, OX-42 and glial fibrillary acidic protein as compared to control rats, suggesting the grape seed extract suppressed sensitization. Another study fed rats diets containing 1% or 10% cocoa, and observed suppression of basal neuronal CGRP expression, in addition to suppression of stimulated iNOS proteins and MAPK p38 [11]. This study confirmed in vivo prior observations from a primary trigeminal ganglia cell model, which secreted less CGRP when pre-treated with a methanol extract of cocoa beans [10]. In humans, meals containing various amounts of macronutrients elicited different responses in plasma CGRP: the high protein meal produced the greatest drop in CGRP, beginning at 30 min post-prandial [31].

A recent publication suggested the link between dietary triggers and oxidative stress [32], including discussion of dietary phenolic “antioxidants”. Anecdotal evidence suggests that chocolate and red wine are migraine triggers, yet we present data on red grape pomace and discuss published results of cocoa research, which demonstrate a decrease of CGRP release in response to in vitro or in vivo exposure to these high-phenolic foods. The dual chemical nature of these molecules, functioning as anti- or pro-oxidants depending on their concentration, may warrant further exploration in relation to migraine. It is possible that one concentration eliciting an antioxidant effect may inhibit migraine, whereas a concentration capable of a pro-oxidant effect may provoke it. Alternatively, it is also interesting to note that this discrepancy bears some resemblance to the CGRP levels observed in relation to medication-overuse headaches (MOH). As mentioned in the Introduction, the triptan family of drugs relieves acute migraine symptoms while also decreasing CGRP levels; however, triptans increase circulating CGRP and cause allodynia in animals with chronic exposure, and cause MOH in humans when taken too frequently [33]. This similarity suggests that the presence of the offending food may not alone precipitate migraine, but the repeated presence of the food followed by its absence may contribute to a rebound effect in sensitive patients.

## 5. Conclusions

CGRP secretion was dramatically and dose-dependently inhibited by treatment with grape pomace extracts, while *S*-petasin and ginger root extract elicited a minor decrease. Meanwhile, at similar doses, grape pomace did not elicit a significant difference in calcium uptake, whereas *S*-petasin and ginger extract inhibited calcium influx. Because of their differing impact on calcium signals, grape pomace extract may impact CGRP release by a different mechanism than ginger extract and *S*-petasin, which inhibit calcium influx to some degree. Overall, if these results were to translate to the in vivo human, the ability of foods to mitigate CGRP release would be considered anti-inflammatory and may decrease occurrence of migraine.

## Figures and Tables

**Table 1:** Maximum change in GCaMP5 ^1^ fluorescence by treatment, relative to vehicle control.

Sample	Treatment Concentration	ΔF/F_o_ (% Vehicle) ^2^	*n*	*P*
Tinta cao extract	1.0 mg PE/mL ^3^	66.2 (55.7)	13	0.146
Cabernet franc extract	1.0 mg PE/mL ^3^	76.8 (43.2)	14	0.365
Ginger extract	0.2 mg equivalent/mL	60.1 (23.9)	14	0.007
*S*-Petasin	10 μM	22.3 (8.4)	6	<0.001

^1^ GCAMP5 is a genetically encoded calcium indicator originated by Janelia Research (Ashburn, VA, USA).

^2^ Data shown as mean (SD); ^3^ PE = pomace equivalents. Vehicle control for all samples was 0.1% DMSO.
